# Multimodal imaging and electrophysiological study in the differential diagnosis of rest tremor

**DOI:** 10.3389/fneur.2024.1399124

**Published:** 2024-05-24

**Authors:** Federica Aracri, Andrea Quattrone, Maria Giovanna Bianco, Alessia Sarica, Marida De Maria, Camilla Calomino, Marianna Crasà, Rita Nisticò, Jolanda Buonocore, Basilio Vescio, Maria Grazia Vaccaro, Aldo Quattrone

**Affiliations:** ^1^Neuroscience Research Center, University “Magna Graecia”, Catanzaro, Italy; ^2^Institute of Neurology, University “Magna Graecia”, Catanzaro, Italy; ^3^Biotecnomed S.c.a.r.l., Catanzaro, Italy

**Keywords:** rest tremor, tremor phase, Parkinson's disease, essential tremor plus, MRI, cortical thickness, machine learning

## Abstract

**Introduction:**

Distinguishing tremor-dominant Parkinson's disease (tPD) from essential tremor with rest tremor (rET) can be challenging and often requires dopamine imaging. This study aimed to differentiate between these two diseases through a machine learning (ML) approach based on rest tremor (RT) electrophysiological features and structural MRI data.

**Methods:**

We enrolled 72 patients including 40 tPD patients and 32 rET patients, and 45 control subjects (HC). RT electrophysiological features (frequency, amplitude, and phase) were calculated using surface electromyography (sEMG). Several MRI morphometric variables (cortical thickness, surface area, cortical/subcortical volumes, roughness, and mean curvature) were extracted using Freesurfer. ML models based on a tree-based classification algorithm termed XGBoost using MRI and/or electrophysiological data were tested in distinguishing tPD from rET patients.

**Results:**

Both structural MRI and sEMG data showed acceptable performance in distinguishing the two patient groups. Models based on electrophysiological data performed slightly better than those based on MRI data only (mean AUC: 0.92 and 0.87, respectively; *p* = 0.0071). The top-performing model used a combination of sEMG features (amplitude and phase) and MRI data (cortical volumes, surface area, and mean curvature), reaching AUC: 0.97 ± 0.03 and outperforming models using separately either MRI (*p* = 0.0001) or EMG data (*p* = 0.0231). In the best model, the most important feature was the RT phase.

**Conclusion:**

Machine learning models combining electrophysiological and MRI data showed great potential in distinguishing between tPD and rET patients and may serve as biomarkers to support clinicians in the differential diagnosis of rest tremor syndromes in the absence of expensive and invasive diagnostic procedures such as dopamine imaging.

## 1 Introduction

Rest tremor (RT) is one of the core clinical signs of Parkinson's disease (PD) occurring in about 75% of PD patients (1, 2), but it can also be found in some non-parkinsonian tremor syndromes, such as Essential Tremor (ET) plus (3, 4). Differentiating tremor-dominant PD (t-PD) from ET with rest tremor (rET) patients can be clinically challenging, especially in tremor-dominant PD phenotype (tPD) or in the early stages of the disease, when bradykinesia and rigidity can be slight or absent (5–7). Single photon emission computed tomography with ^123^I-ioflupane (DaTscan) has a crucial role in differentiating parkinsonian RT from other tremulous diseases (4, 8, 9), but it is an expensive, invasive and time-consuming procedure not widely available and not commonly employed in the clinical routine (4). In the last years, the electrophysiological examination of RT has gained a growing importance in the differential diagnosis of rest tremor syndromes (10, 11). It is a cheap and available procedure which can be easily performed worldwide and can support the clinical diagnosis (10–17). In addition to neurophysiology, brain MRI availability has significantly increased over the last decades and it is now often included in the diagnostic work-up of patients with tremor, with the primary aim of ruling out structural causes (4). A few pilot studies showed high potential of MRI quantitative data in distinguishing PD from rET patients using a machine learning approach based on MR volumes and diffusion tensor imaging (DTI) data of basal ganglia and cerebellum (18), or metabolic alterations in the thalamus detected by MR spectroscopy (19). In addition, a recent functional MRI study found differences between PD and rET also in cortical regions (20). The exact role of advanced MRI in distinguishing between these two diseases, however, is still largely unknown and needs further investigations. Recently, machine learning (ML) algorithms have been successfully applied to distinguish among different neurological disorders (21–28), and the combination of different sources of data (i.e., clinical, imaging, electrophysiological, and fluid) often provided the best classification performance (25, 29).

In the current study, we employed a modern powerful ML decision tree-based classification algorithm [eXtreme Gradient Boosting [XGBoost] (30)] to compare the classification performance of structural MR imaging data (several cortical metrics and subcortical volumes) with those of the RT electrophysiological features in distinguishing between rET and tPD patients. Furthermore, we investigated whether the combination of electrophysiological and imaging data may improve the classification performance.

## 2 Materials and methods

### 2.1 Study participants

Forty tPD and 32 rET consecutive patients with available 3T brain MRI and surface electromyographic (sEMG) analysis of RT, and 45 age- and sex-matched control subjects (HC) were included in this study. The clinical diagnoses of rET (now included in the “ET plus” category) and PD were performed by a movement disorder specialist in accordance with the recent consensus statement of the Movement Disorder Society task force (3) and international diagnostic criteria for PD (2). Only PD patients classified as “tremor-dominant” using the MDS-UPDRS sub-scores (assessment performed in OFF state) as previously described (31) were included in the study. All patients underwent a detailed neurological examination, a sEMG analysis of the rest tremor, a brain 3T-MRI scan, and a 123I-FP-CIT-SPECT (DaTscan) performed as described previously (17) and in Supplementary material, with visual assessment and semi-quantitative analysis through DaTQUANT (32). The brain MRI and sEMG tremor analysis were performed on the same day of the clinical examination, and the DaTscan was performed within 1 month. All study procedures and ethical aspects were approved by the institutional review board (Magna Graecia University review board, Catanzaro, Italy). Written informed consent for the research was obtained from all the individuals participating in the study. Further information and detailed exclusion criteria in Supplementary material.

### 2.2 Electrophysiological examination

All patients underwent a sEMG recording in the most affected upper limb with RT, as described in previous publications (12, 17) and in Supplementary material. The patient was seated in a comfortable chair with the arm flexed at 90°, the forearm fully supported against gravity, and the hand hanging down from the chair armrest. All drugs that might interfere with tremor were suspended 2 days before the examination. The bursts were manually segmented from the filtered sEMG signals, and the mean burst amplitude was evaluated. Spectral analysis was performed to extract the frequency and signal-to-noise ratio associated with the tremor peak. Quantitative RT phase values were determined using cross-spectral analysis, as described by Boose et al. (33), as an indicator of the temporal relationship between extensor and flexor bursts. A phase close to 0° corresponds to synchronous extensor and flexor signals, while a phase close to 180° describes a temporal shift between the contraction bursts of antagonistic muscles. The sEMG examination was coupled with simultaneous acquisition of inertial data in all patients through an accelerometer positioned on the hand with tremor at rest.

### 2.3 MRI acquisition

All MRI scans were performed with the same 3-T MR750 General Electric scanner with an 8-channel head coil (Discovery MR- 750, GE, Milwaukee, WI, USA). The acquisition protocol is detailed in Supplementary material.

### 2.4 MR image processing and feature extraction

The automated neuroanatomical segmentation was performed with FreeSurfer 7.1.1 software (Massachusetts General Hospital, Harvard Medical School; http://surfer.nmr.mgh.harvard.edu), on T1-weighted MR images in all study participants. Structural images were automatically processed with the standard pipeline (recon-all), including: motion correction and averaging volumetric T1 weighted images; removal of non-brain tissue; automated Talairach transformation; segmentation of the subcortical white matter and deep gray matter volumetric structures; tessellation of the gray matter white matter boundary; automated topology correction; surface deformation to optimally place the GM/WM and GM/cerebrospinal fluid (CSF) borders at the location (34). We employed advanced surface-based and volume-based techniques capable of estimating multiple complementary morphometric characteristics of cortical structures, and the following morphometric metrics were calculated into 34 cortical regions of interest (ROIs) per hemisphere according to the Desikan–Killiany atlas: cortical thickness, surface area, cortical volume, mean curvature and roughness (the standard deviation of cortical thickness) (23). Subcortical structures (cerebellum, thalamus, caudate, putamen, globus pallidus, hippocampus, amygdala, and nucleus accumbens) were also segmented to obtain volumetric data. A total of 358 structural features were extracted from each subject.

### 2.5 Statistical analysis

Difference in sex distribution was assessed with Fisher's exact test. Normality of data was tested using Shapiro's test. The analysis of variance (ANOVA) or Kruskal-Wallis test were employed for comparing age at examination among the three groups (tPD, rET, and HC). Age at disease onset, disease duration, RT duration and electrophysiological data were compared between the two patient groups using *t*-test or Wilcoxon rank sum test. ANCOVA with age as covariate was applied to assess differences in MMSE. ANCOVA with age and sex as covariates was used to compare the cortical metrics among groups. ANCOVA with age, sex and total intracranial volume (TIV) as covariates was used to compare the subcortical volumes among groups. All tests were two tailed, and the α level was set at *p* < 0.05. All *p*-values were corrected according to Bonferroni. Statistical analysis was conducted with R language version 4.1.2.

### 2.6 Machine learning models

#### 2.6.1 ML model development

We investigated the performances of machine learning (ML) models using an algorithm termed XGBoost (30) based on different combinations of structural MR imaging data and or RT electrophysiological features in distinguishing between t-PD and rET patients. First, we investigated the performances of ML models using either MRI or RT electrophysiological data separately; subsequently, we developed models combining imaging and sEMG data, aiming to improve the classification performance. The analyses were conducted with Python 3.9 and the packages scikit-learn v1.0.2. The RT electrophysiological features included RT amplitude, frequency and phase; the imaging features included, for each of the 68 cortical regions, 34 for each hemisphere: cortical thickness, cortical volumes, surface areas, mean curvature and roughness, and subcortical volumes. For each XGBoost model, the hyperparameters [learning rate, maximum depth, minimum child weight, gamma, and colsample bytree (the fraction of features used to train each tree)] of the ML models were tuned through 5-fold cross-validation (5-fold cv) repeated 5 times, with randomized search (100 iterations) to maximize the accuracy.

#### 2.6.2 Feature selection procedure

To reduce the number of features which may bring redundant information and noise in the data, we used a two-step feature selection procedure. We first calculated the feature importance with the “permutation accuracy importance” technique (35) assessing the Mean Decrease in Accuracy after permuting each feature, using 50 repetitions to ensure the reliability of the feature ranking. Feature selection was then applied by iteratively training the models on the variables ordered according to the permutation importance.

#### 2.6.3 Classification performance

The performance of the XGB models trained on the most important features were evaluated in cross-validation (cv) analysis, though two alternative cv approaches (repeated stratified 5-fold cross cv and leave-one out). First, the dataset was divided into K subsets (folds) and the models were iteratively trained K times. For each iteration, each model was trained on (K-1) folds as described above and the performance was assessed in the Kth (validation) fold, which was not used for model training, to ensure performance assessment on unseen data. The mean and standard deviation of area under the curve (AUC), accuracy, sensitivity, and specificity in validation folds were calculated. A model was considered able to distinguish between groups when the mean AUC in the validation folds was >0.85. The performances were compared across different models using Friedman test followed by the Durbin-Conover *post-hoc* for pairwise comparisons.

## 3 Results

### 3.1 Patients

Demographic, clinical and electrophysiological data of patients and controls are shown in Table 1. No differences were found in age and sex among groups. tPD patients had shorter disease duration than rET patients but the two groups had similar duration of tremor at rest. tPD patients had slight cognitive impairment in comparison with control subjects, but no significant difference was found between the two patient groups. Regarding electrophysiological data, the RT was characterized by higher amplitude, higher phase values and lower frequency in tPD patients than in rET patients (Table 1). sEMG-derived amplitude showed a strong Pearson's linear correlation (*r*: 0.841; *p* < 0.001) with accelerometric amplitude of RT. The DaTscan was normal in all rET patients and abnormal in all PD, per inclusion criteria. All PD patients had reduced DAT uptake in the putamen contralateral to the clinically most affected side (with or without milder involvement of the ipsilateral putamen and the caudate nuclei; Supplementary Table 1).

**Table 1:** Demographic, clinical and electrophysiological data of patients with Parkinson's disease, patients with essential tremor with rest tremor, and control subjects.

**Data**	**Tremor dominant PD**	**ET with rest tremor**	**Control subjects**	***p*-value**
	**(*N* = 40)**	**(*N* = 32)**	**(*N* = 45)**	
Sex (M/F)	21/19	14/18	27/18	0.369^a^
Age at examination, years^b^	66.2 ± 8.8	64.4 ± 11.0	68.5 ± 6.9	0.341^c^
Age at disease onset, years^b^	62.5 ± 8.5	45.1 ± 15.0	/	**< 0.001** ^**d**^
Disease duration, years^b^	3.7 ± 3.4	16.8 ± 11.8	/	**< 0.001** ^**d**^
RT duration, years^b^	3.7 ± 3.4	4.7 ± 3.9	**/**	0.270^d^
MDS-UPDRS-III score^b^	23.0 ± 14.4	/	/	/
H-Y score^b^	1.4 ± 0.6	**/**	**/**	**/**
MMSE^b^	23.2 ± 5.1^**^	25.6 ± 3.7	27.6 ± 2.1	**< 0.001** ^**e**^
**Electrophysiological data**				
RT amplitude^b^	189.6 ± 99.8	76.7 ± 67.7	/	**< 0.001** ^**d**^
RT frequency^b^	5.0 ± 0.7	5.6 ± 1.0	/	**0.003** ^**d**^
RT phase^b^	126.2 ± 54.3	26.7 ± 37.0	**/**	**< 0.001** ^**d**^

PD, Parkinson's disease; ET, essential tremor; RT, rest tremor; MDS-UPDRS-III, Movement Disorder Society—Unified Parkinson's Disease Rating Scale- part III (Motor Examination); H-Y, Hoehn and Yahr; MMSE, Mini Mental State Examination.

Significant p-values are in bold.

^a^Fisher's exact test.

^b^Data are expressed as mean ± standard deviation.

^c^ANOVA or Kruskal-Wallis rank sum test, followed by t-test or Wilcoxon rank sum test with Bonferroni correction.

^d^t-test or Wilcoxon rank sum test.

^e^ANCOVA among the three groups with age as covariate, followed by *post-hoc* with Bonferroni correction.

^**^Tremor-dominant PD vs. controls, p < 0.001.

### 3.2 MRI cortical and subcortical morphometric features

Tremor-dominant PD patients showed increased mean curvature in the right inferior temporal gyrus, increased roughness in the left isthmus cingulate cortex and an increased cortical thickness in bilateral orbito-frontal regions in comparison with control subjects (Supplementary Table 2). ET patients with rest tremor showed increased roughness and mean curvature in temporal regions while these metrics were reduced in a few other regions in comparison with controls (Supplementary Table 2).

By directly comparing the two patient groups, rET showed reduced cortical thickness in the right medial orbitofrontal cortex and in the left lateral occipital cortex in comparison with tPD.

No differences were found in the volumes of subcortical structures among the three groups.

### 3.3 Machine learning models

Among ML models based on imaging data, we first assessed the performance of models using one structural MRI metric at a time. The model using cortical thickness was the most powerful one though showing a suboptimal AUC of 0.788 ± 0.109 in distinguishing between tPD and rET patients; none of the other models reached AUC values above 0.80. By employing as input for the models different structural MRI metrics together, the classification performance improved and the best results (AUC: 0.868 ± 0.086) were achieved by a model using a combination of mean curvature and roughness (Table 2).

**Table 2:** Classification performances of XGBoost models based on rest tremor electrophysiological features and structural MRI features in distinguishing between patients with tremor-dominant Parkinson's disease and patients with essential tremor with rest tremor.

**Cortical thickness**	**Cortical volumes**	**Surface area**
AUC: 0.788 (0.109)	AUC: 0.655 (0.103)	AUC: 0.655 (0.119)
Acc: 0.708 (0.097)	Acc: 0.588 (0.076)	Acc: 0.591 (0.106)
Sens: 0.775 (0.132)	Sens: 0.900 (0.137)	Sens: 0.690 (0.146)
Spec: 0.626 (0.141)	Spec: 0.207 (0.217)	Spec: 0.463 (0.188)
(#7)	(#6)	(#2)
**Subcortical volumes**	**Mean curvature**	**Roughness**
AUC: 0.503 (0.131)	AUC: 0.653 (0.135)	AUC: 0.542 (0.151)
Acc: 0.520 (0.095)	Acc: 0.605 (0.116)	Acc: 0.512 (0.148)
Sens: 0.655 (0.188)	Sens: 0.920 (0.105)	Sens: 0.645 (0.186)
Spec: 0.350 (0.209)	Spec: 0.220 (0.248)	Spec: 0.349 (0.189)
(#1)	(#14)	(#31)
**RT amplitude**	**RT frequency**	**RT phase**
AUC: 0.797 (0.104)	AUC: 0.736 (0.121)	AUC: 0.880 (0.079)
Acc: 0.764 (0.098)	Acc: 0.747 (0.099)	Acc: 0.826 (0.072)
Sens: 0.835 (0.116)	Sens: 0.875 (0.132)	Sens: 0.815 (0.112)
Spec: 0.673 (0.143)	Spec: 0.589 (0.142)	Spec: 0.840 (0.162)
**Best MR model**	**Best sEMG model**	**Best combined model**
AUC: 0.868 (0.086)	AUC: 0.924 (0.053)	AUC: 0.967 (0.032)
Acc: 0.779 (0.111)	Acc: 0.845 (0.076)	Acc: 0.892 (0.069)
Sens: 0.815 (0.123)	Sens: 0.850 (0.106)	Sens: 0.920 (0.093)
Spec: 0.734 (0.151)	Spec: 0.838 (0.167)	Spec: 0.854 (0.138)
(#9)	(#2)	(#6)

AUC, Area Under the Curve; Acc, Accuracy; Sens, sensitivity; Spec, specificity; MR, Magnetic Resonance; RT, rest tremor; sEMG, surface electromyography.

Data are shown as mean (standard deviation) in the repeated 5-fold cross-validation folds. The number of features used by each model determined using feature selection is reported in round brackets (#).

The best MR model included mean curvature and roughness.

The best sEMG model included RT amplitude and phase.

The best combined model used as input the cortical volumes, surface areas, mean curvature, RT amplitude and RT phase and effectively used mean curvature, RT amplitude and RT phase after feature selection.

Among the different RT electrophysiological features, the best model in distinguishing between t-PD and rET patients (AUC: 0.924 ± 0.053) used a combination of RT phase and amplitude; slightly lower performances were obtained using the RT phase alone (AUC: 0.880 ± 0.079), followed by RT amplitude (AUC: 0.797 ± 0.104) and RT frequency (AUC: 0.736 ± 0.121; Table 2).

**Figure 1:**
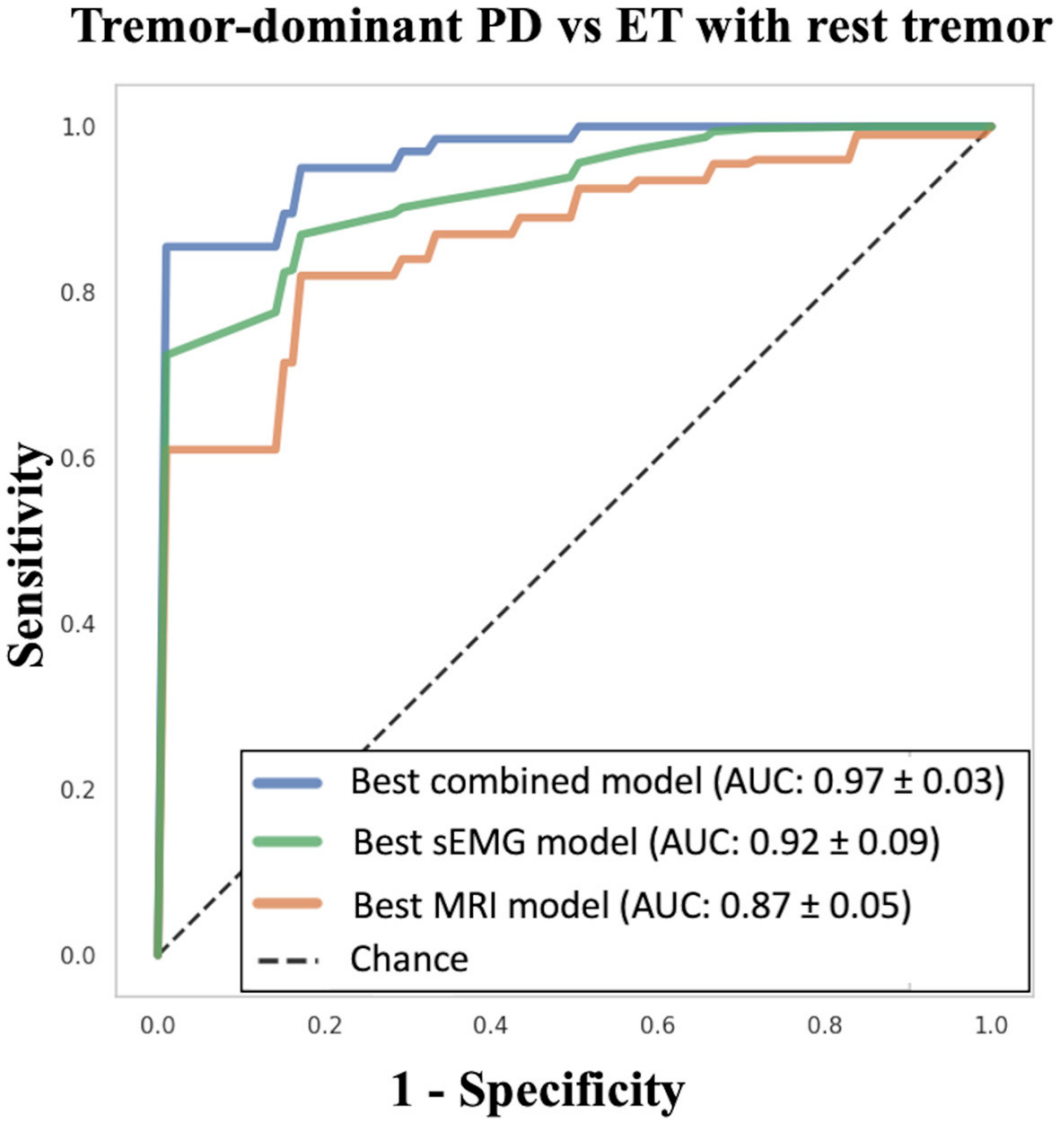
Machine learning models in differentiating between tPD and rET patients. The XGBoost “best combined model” was based on RT amplitude, RT phase and mean curvature. The XGBoost “best sEMG model” was based on RT amplitude and phase. The XGBoost “best MRI model” was based on mean curvature and roughness. tPD, tremor-dominant Parkinson's disease; rET, essential tremor with rest tremor; RT, rest tremor; sEMG, surface electromyography; AUC, Area Under the Curve; SD, standard deviation.

**Figure 2:**
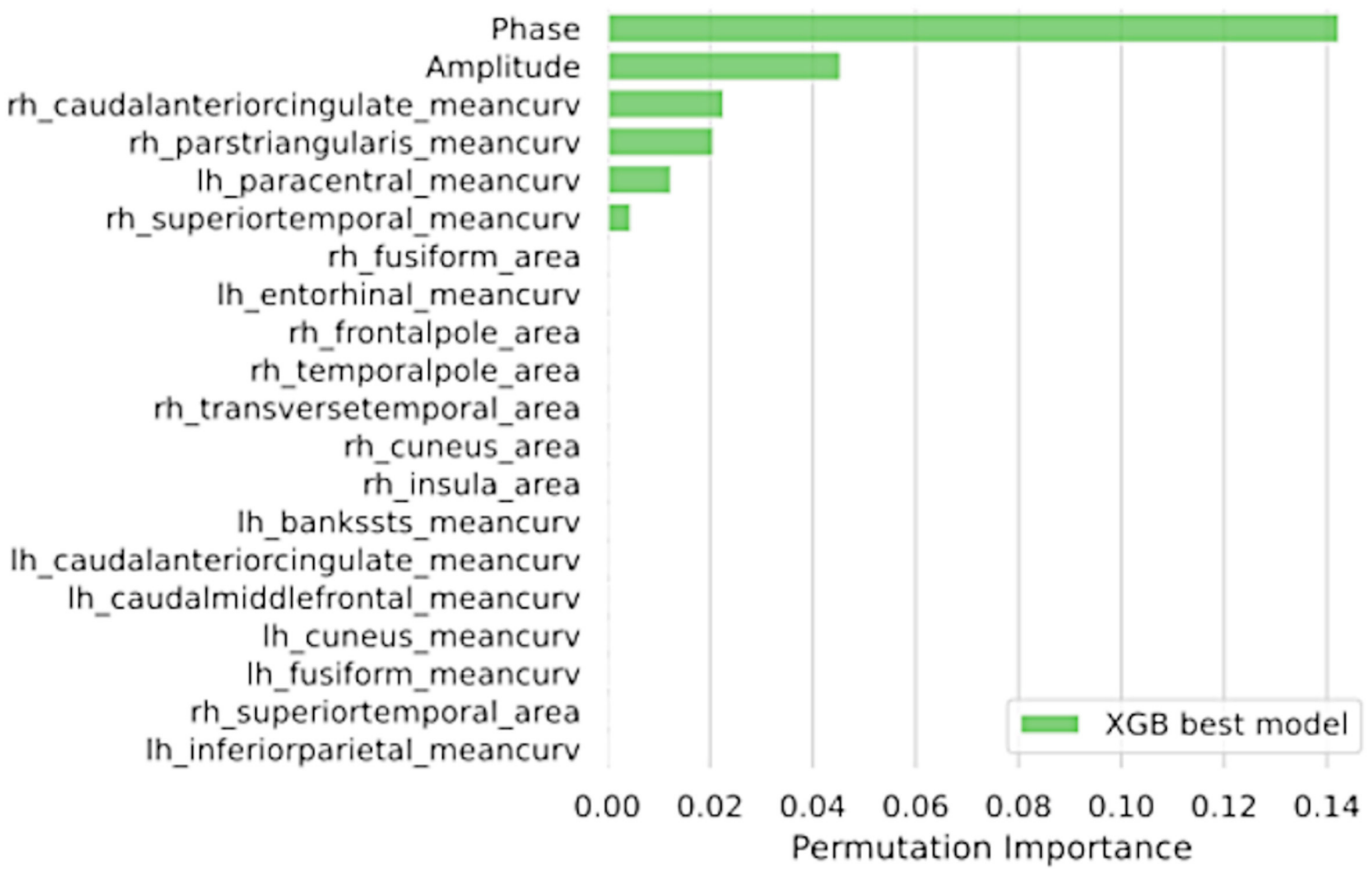
The feature importance assessed via permutation methods of the best combined XGBoost model including both electrophysiological and MRI data in distinguishing between between tPD and rET patients. Data are shown in descending order from the most to the less important feature. tPD, tremor-dominant Parkinson's disease; rET, essential tremor with rest tremor; Rh, right; Lh, left.

Overall, the best ML models using either structural MRI data or sEMG data showed acceptable classification performances (AUC > 0.85) in distinguishing between tPD and rET patients. By comparing the two data sources, the best ML model based on RT electrophysiological data yielded higher performance (AUC: 0.924 ± 0.053; sensitivity: 85.0%, specificity: 83.8%, accuracy: 84.5%) in distinguishing tPD from rET patients than the best model based on MR structural data only (AUC: 0.868 ± 0.086; sensitivity: 81.5%, specificity: 73.4%, accuracy: 77.9%; *p* = 0.0071; Table 2 and Supplementary Table 3). In this study, the top-performing model in discriminating between rET and tPD (AUC: 0.967 ± 0.032), however, included a combination of sEMG and MRI data, outperforming models using the two data sources separately (Figure 1, Table 2, and Supplementary Table 3). The best model showed all classification metrics above 85%, with sensitivity: 92.0%, specificity: 85.4%, and accuracy: 89.2% (Table 2). In this model, the feature importance analysis identified the RT phase as the most informative feature for classification between the two groups, followed by RT amplitude and mean curvature (Figure 2). Almost identical results were obtained with leave-one-out cross-validation procedures (Supplementary Table 4).

## 4 Discussion

In this study, machine learning models using structural MRI data and RT electrophysiological features showed great potential in discriminating between t-PD and rET patients.

Differentiating between tremor-dominant Parkinson's disease and non-parkinsonian rest tremor syndromes can be really challenging, and ancillary diagnostic tests are useful to support the clinical diagnosis (3–5, 7, 8). The gold standard diagnostic procedure in this context is the DaTscan, which typically reveals striatal dopaminergic deficit in PD patients and integrity of the dopaminergic system in other RT syndromes such as ET plus (4, 8, 9). Dopamine imaging, however, is expensive, invasive and not widely available, and there is thus a need for other biomarkers to distinguish between these tremulous disorders. These tests may include tremor analysis (with accelerometry or surface EMG), smell testing, transcranial sonography and brain MRI. We have recently demonstrated that the RT electrophysiological analysis could be used as surrogate biomarker of dopamine imaging in patients with RT (17); on the other hand, smell testing and sonography showed some potential in distinguishing PD from ET patients (36, 37), but only few data exist in ET patients with rest tremor (38), and the role of structural MRI in distinguishing between tPD and rET remains largely unexplored.

In this study, we investigated differences between t-PD and rET patients in multiple MRI cortical morphometric measures (thickness, volume, surface area, mean curvature, and roughness) and subcortical volumes, all derived from T1-weighted images. Despite the large amount of considered features, the two patient groups diverged only in the thickness of a couple of cortical regions (right medial orbitofrontal and left lateral occipital gyrus), which was slightly lower in rET than in t-PD patients, demonstrating that only minimal structural differences exist between these tremulous disorders.

Interestingly, a powerful machine learning decision-tree-based ensemble algorithm using eXtreme Gradient Boosting (XGBoost), was able to discriminate with acceptable performances (AUC: 0.868 ± 0.086) between these two diseases by using a combination of cortical metrics, demonstrating that machine learning technology can take advantage of subtle differences and combine them together to achieve good classification performances. Indeed, this ML algorithm builds a sequential ensemble of trees with the aim to improve the performance of the previous tree by correcting its errors, thus having the ability to learn from its wrong predictions, which are corrected by giving more weight to the misclassified instances, thus leading to high classification accuracy (39). We first assessed the performance of ML models using structural MRI metrics separately (i.e., thickness only, roughness only, etc…). The model based on cortical thickness was the most powerful, in agreement with the slight group differences in cortical thickness between PD and rET patients, but none of these models reached AUC values above 0.80. The performance improved when different cortical metrics were used together as input for the models, demonstrating that surface-based and volume-based metrics can explore complementary morphometric characteristics of cortical structures, detecting even subtle cortical alterations.

In the current study, we also compared the classification performances of brain MRI data with those of electrophysiological RT features, using the same ML algorithm. RT showed higher amplitude, higher phase values and lower frequency in PD patients than in rET patients, in agreement with the existing literature (12, 16, 17). Machine learning models employing a combination of electrophysiological RT features distinguished the two patient groups with AUC of 0.924 ± 0.053 using RT phase as the most important feature, slightly outperforming models based on MRI data. The higher phase values of RT tremor in PD than in rET patients reflects the empirical observation of alternating muscular contraction pattern in PD and synchronous pattern in rET patients. It is possible that different tremor generators are involved in these two conditions, and a possible dystonic basis of RT in rET patients has been hypothesized to explain the co-contracting pattern (40), but the exact pathophysiological bases of the different RT patterns across PD and rET remain to be determined.

Finally, we combined MRI metrics and RT electrophysiological features into ML models, leading to an extremely high classification performances (AUC: 0.967 ± 0.032) in distinguishing between these two patient groups. This result demonstrates a complementary role of these two diagnostic procedures (advanced brain MRI and RT electrophysiological analysis), both of whom can be helpful in the differential diagnosis of rest tremor syndromes, especially when combined together. Our ML model also provided insights on the measures that helped most in the classification of patients, by automatically selecting the RT phase as the feature with the highest importance score, followed by RT amplitude and then by MRI metrics, confirming the relative superiority of sEMG over structural MRI in this classification task. Our combined approach has the obvious advantage of yielding higher diagnostic performances, and the relative drawback of requiring different sources of data (sEMG and MRI); these techniques, however, are available and often included in the diagnostic work-up of tremor, thus not requiring additional tests for patients.

The current study is one of the first MR imaging studies comparing tremor-dominant PD and rET patients, and has several strengths. First, we compared the performance of ML models based exclusively on either MRI or sEMG data and on a combination of the two data sources, to get objective insights on the diagnostic usefulness of these two different procedures. Second, differently from most studies which focused on the differential diagnosis between PD and classical ET syndrome, we included in the study only patients ET patients with tremor at rest (rET), whose differential diagnosis represents a higher clinical challenge. Finally, to minimize potential misdiagnosis and thus increase the reliability of our findings, all patients included in the study had the clinical diagnosis supported by DaTscan (abnormal with a typical pattern in tPD patients and normal in rET patients).

The main limitations to this study are the relatively limited sample size and the lack of an independent validation cohort. In this study, the performances of the ML models were assessed using 5-fold cross-validation with 5 repetitions thus calculating the mean AUC on unseen data (data not used to train the models) and increase the reliability of the findings. However, future studies to validate the performances of these models based on structural MR data and sEMG in independent patient cohorts are needed. Second, a relative limitation is the limited explainability of gradient boosting machines when it comes to individual predictions (30). We employed the model-agnostic permutation approach to provide information on the features contributing most to the prediction. Although this is a robust and widely used approach to assess the impact of a feature on the model's performance (35), it describes the global behavior of the model without considering interactions among features. Recent explainable machine learning algorithms can provide information on the features used for prediction at the individual level and may represent a step toward increasing the confidence of clinicians with ML models. Finally, a limitation to the immediate widespread use of such biomarkers is the complexity of ML approaches, which require high-level expertise and technology and are thus not yet suitable for clinical routine; however, there is a huge interest in ML use in the medical field for diagnostic and prognostic purposes, making these approaches likely available in clinical practice in the near future through automatized and user-friendly tools. In this context it is of high relevance the source of data included into the machine learning models, which should not contribute limiting the feasibility of such ML approaches. In this study, we developed an extremely accurate model including only electrophysiological data and structural MRI data obtained from T1-weighted images, without any specific MR sequence and any nuclear medicine procedure, hopefully allowing a larger use of such biomarkers. Future studies may investigate the usefulness of combining electrophysiology with other bedside testing (i.e., sniffing tests, Rem Behavior Disorder Questionnaires, or brain sonography), or with specific MRI techniques such as iron-sensitive MRI, in differentiating among rest tremor syndromes.

## 5 Conclusion

In conclusion, this is the first study combining MRI data and RT electrophysiological data to accurately distinguish between rET and tPD patients *in vivo*. This finding, if further validated into independent cohorts, may have a positive impact on tremor diagnosis, also translating into economic advantages by reducing the use of expensive procedures such as dopamine imaging.

## Data availability statement

The raw data supporting the conclusions of this article will be made available by the authors, without undue reservation.

## Ethics statement

The studies involving humans were approved by Institutional Review Board (Magna Graecia University Review Board, Catanzaro, Italy). The studies were conducted in accordance with the local legislation and institutional requirements. The participants provided their written informed consent to participate in this study.

## Author contributions

FA: Conceptualization, Data curation, Formal analysis, Writing – original draft. AnQ: Conceptualization, Supervision, Writing – original draft, Writing – review & editing. MB: Data curation, Formal analysis, Writing – review & editing. AS: Formal analysis, Methodology, Supervision, Writing – review & editing. MD: Data curation, Writing – review & editing. CC: Formal analysis, Methodology, Writing – review & editing. MC: Data curation, Writing – review & editing. RN: Data curation, Writing – review & editing. JB: Data curation, Writing – review & editing. BV: Data curation, Methodology, Supervision, Writing – review & editing. MV: Data curation, Writing – review & editing. AlQ: Conceptualization, Supervision, Writing – review & editing.
