# Supplementary material for: Multimodal imaging and electrophysiological study in the differential diagnosis of rest tremor
Source: Front Neurol. 2024 May 24;15:1399124. doi: 10.3389/fneur.2024.1399124 (PMC11160119; doi:10.3389/fneur.2024.1399124)
Supplement: Supplementary file 1 [file Table_1.DOCX]

**Supplementary Table 1.** Dopamine imaging data of patients with Parkinson’s disease and patients with essential tremor with rest tremor.

| **Data** | **Tremor dominant PD**  **(N=40)** | **ET with rest tremor**  **(N=32)** | **p value** |
| --- | --- | --- | --- |
| *Dopamine imaging data (DaTscan)* |  |  |  |
| Caudate uptake contralateral to the MAS^a^ | 1.44 ± 0.57 | 2.52 ± 0.50 | < 0.001^b^ |
| Caudate uptake ipsilateral to the MAS^a^ | 1.65 ± 0.50 | 2.59 ± 0.52 | < 0.001^b^ |
| Putamen uptake contralateral to the MAS^a^ | 0.70 ± 0.24 | 2.18 ± 0.33 | < 0.001^b^ |
| Putamen uptake ipsilateral to the MAS^a^ | 0.99 ± 0.34 | 2.19 ± 0.30 | < 0.001^b^ |
| Pattern of DaTSCAN abnormality (UP/UCP/BP/BCP) | 8/1/18/13 | 0/0/0/0 | **/** |

Abbreviations: PD = Parkinson’s disease; ET = essential tremor; MAS = clinically most affected side; the MAS was the clinical side with rest tremor (if unilateral) or with the most severe rest tremor (if present bilaterally); UP = unilateral involvement of the putamen (always contralateral to the clinically most affected side); UCP = unilateral involvement of the caudate and putamen; BP = bilateral involvement of the putamina; BCP = bilateral involvement of the putamina and either unilateral or bilateral involvement of the caudate nuclei. The mean z-scores from controls (according to the DATQUANT normative values) were: -3.26 for putamen contralateral to the MAS, -2.48 for putamen ipsilateral to the MAS, -1.66 for caudate contralateral to the MAS, -1.08 for caudate ipsilateral to the MAS.

Significant p values are in bold.

^a^Data are expressed as mean ± standard deviation.

^b^ANCOVA among groups with age as covariate.
