# Supplementary material for: Multimodal imaging and electrophysiological study in the differential diagnosis of rest tremor
Source: Front Neurol. 2024 May 24;15:1399124. doi: 10.3389/fneur.2024.1399124 (PMC11160119; doi:10.3389/fneur.2024.1399124)
Supplement: Supplementary file 2 [file Table_2.DOCX]

**Supplementary Table 2.** Significant differences in MRI metrics among patients with tremor-dominant Parkinson’s disease, patients with essential tremor with rest tremor, and control subjects.

| **Regions** | **Lobe** | **tPD** | **rET** | **Controls** | **p value** | **Pairwise comparisons** |
| --- | --- | --- | --- | --- | --- | --- |
| ***CORTICAL THICKNESS*** |  |  |  |  |  |  |
| Rh medial orbitofrontal | Frontal | 2.502 (0.021) | 2.423 (0.024) | 2.467 (0.020) | 0.043 | rET< tPD |
| Lh lateral occipital | Occipital | 2.126 (0.016) | 2.057 (0.018) | 2.078 (0.016) | 0.019 | rET < tPD |
| Lh lateral orbito-frontal | Frontal | 2.835 (0.024) | 2.793 (0.027) | 2.736 (0.023) | 0.010 | tPD > HC |
| Rh lateral orbito-frontal | Frontal | 2.748 (0.023) | 2.719 (0.027) | 2.667 (0.023) | 0.042 | tPD > HC |
| Rh postcentral | Parietal | 2.042 (0.016) | 2.071 (0.018) | 2.011 (0.016) | 0.048 | rET > HC |
| ***CORTICAL ROUGHNESS*** |  |  |  |  |  |  |
| Lh isthmus cingulate | Parietal | 0.776 (0.010) | 0.747 (0.012) | 0.732 (0.010) | 0.007 | tPD > HC |
| Rh superior temporal | Temporal | 0.605 (0.008) | 0.615 (0.009) | 0.584 (0.007) | 0.025 | rET > HC |
| ***MEAN CURVATURE*** |  |  |  |  |  |  |
| Rh inferior temporal | Temporal | 0.135 (0.001) | 0.133 (0.001) | 0.130 (0.001) | 0.010 | tPD > HC |
| Lh precuneus | Parietal | 0.123 (0.001) | 0.125 (0.001) | 0.126 (0.001) | 0.041 | tPD < HC |
| Rh rostral-anterior cingulate | Frontal | 0.132 (0.002) | 0.125 (0.003) | 0.137 (0.002) | 0.003 | rET < HC |
| Lh Banks of the Superior Temporal Sulcus | Temporal | 0.096 (0.002) | 0.101 (0.002) | 0.094 (0.002) | 0.025 | rET > HC |
| Lh transverse temporal | Temporal | 0.108 (0.002) | 0.101 (0.003) | 0.112 (0.002) | 0.015 | rET < HC |
| Rh cuneus | Occipital | 0.128 (0.002) | 0.126 (0.002) | 0.133 (0.001) | 0.015 | rET < HC |
| ***CORTICAL AREAS*** |  |  |  |  |  |  |
| Lh isthmus cingulate | Parietal | 958.15 (22.01) | 909.47 (24.86) | 877.89 (21.09) | 0.029 | tPD > HC |

Abbreviations: tPD = tremor dominant Parkinson’s disease; rET = Essential Tremor with rest tremor.

Data are the mean values in each region, adjusted for covariates (age and sex), expressed in mm for thickness and roughness, and in mm^-1^ for mean curvature. Data shown in brackets refer to standard error. Only the metrics significantly different among groups are shown in the table. Statistical comparisons were performed using ANCOVA on raw values with age and gender as covariates, followed by post-hoc pairwise comparisons with Bonferroni correction.
