# Supplementary material for: Multimodal imaging and electrophysiological study in the differential diagnosis of rest tremor
Source: Front Neurol. 2024 May 24;15:1399124. doi: 10.3389/fneur.2024.1399124 (PMC11160119; doi:10.3389/fneur.2024.1399124)
Supplement: Supplementary file 3 [file Table_3.DOCX]

**Supplementary Table 3.** Statistical differences among the performances of different XGBoost models in distinguishing between patients with tremor-dominant Parkinson’s disease and patients with essential tremor with rest tremor

| **Data** | **Best combined model** | **Best MR**  **model** | **Best sEMG model** | **RT phase** |
| --- | --- | --- | --- | --- |
| **Best combined model** | - | p = 0.0001 | p = 0.0231 | p = 0.0008 |
| **Best MR model** | - | - | p = 0.0071 | - |

The best MR model included mean curvature and roughness.

The best sEMG model included RT amplitude and phase.

The best combined model used as input the cortical volumes, surface areas, mean curvature, RT amplitude and RT phase and effectively used mean curvature, RT amplitude and RT phase after feature selection.

The performances were compared across different models using Friedman test followed by the Durbin-Conover post-hoc for pairwise comparisons.
