# Supplementary material for: Multimodal imaging and electrophysiological study in the differential diagnosis of rest tremor
Source: Front Neurol. 2024 May 24;15:1399124. doi: 10.3389/fneur.2024.1399124 (PMC11160119; doi:10.3389/fneur.2024.1399124)
Supplement: Supplementary file 4 [file Table_4.DOCX]

**Supplementary Table 4:** Classification performances of XGBoost models based on rest tremor electrophysiological features and structural MRI features in distinguishing between patients with tremor-dominant Parkinson’s disease and patients with essential tremor with rest tremor, by using leave-one-out cross-validation procedures.

|  | **Best MR model** | **Best sEMG model** | **Best combined model** |
| --- | --- | --- | --- |
| AUC: | 0.840 | 0.903 | 0.966 |
| Accuracy: | 0.736 | 0.847 | 0.888 |
| Sensitivity: | 0.800 | 0.875 | 0.925 |
| Specificity: | 0.656 | 0.812 | 0.843 |

Abbreviations: AUC = Area Under the Curve, MR = Magnetic Resonance; sEMG = surface electromyography.
